# Co-developing a Mental Health and Wellbeing Chatbot With and for Young People

**DOI:** 10.3389/fpsyt.2020.606041

**Published:** 2021-02-01

**Authors:** Christine Grové

**Affiliations:** Educational Psychology and Inclusive Education Academic Community, Monash University, Melbourne, VIC, Australia

**Keywords:** mental health, youth, digital intervention, artificial intelligence, chatbot

## Abstract

There are many young people who experience mental health and wellbeing challenges. A potential negative mental health trigger for some youth is a struggle to cope with stress at school, feelings of depression and anxiety and availability of adequate help for these stressors. In response to youth needs a mental health and wellbeing Chatbot has been co-developed with youth, technology partners and expert stakeholders. An element of the Chatbot is powered by artificial intelligence and rules based AI using natural language processing. It is created to communicate evidence based resources, wellbeing support, educational mental health information and adaptive coping strategies. This paper will discuss how the Chatbot has been developed, highlighting its participatory, co-design process with youth who are the key stakeholders to benefit from this digital tool. Research from interviews and surveys informed the creation of the Chabots personality and its character design. Examples of the conversation design and content development are provided. The paper finishes with how, if at all, digital tools such as Chatbot applications could support the mental health of young people in secondary schools or health care settings in conjunction with the wellbeing or health care team, concluding with lessons learned and cautions.

## Introduction

Almost a quarter of young people aged 15–19 have symptoms of serious mental illness (1). This figure is increasing. With modelling suggesting that mental illness may increase as a result of COVID-19, particularly amongst young people (2). Key stressors for youth are: coping with stress; school and study problems; and mental health challenges. Top issues also include experiences of inequality and discrimination (1). Young people who experience mental health difficulties may disengage from their schooling or the community through absenteeism, being socially isolation or financially disadvantaged. They also may be considered at risk due to geographical location (rural), ethnic and/or racial ethnicity, sexual identity, having a disability and/or their family background. The World Health Organization (3) recently announced that depression is now the leading cause of ill health and disability, having risen by more than 18 per cent worldwide since 2005. This paper will address the significant need to provide youth with accessible wellbeing support that they need and want to access.

Mental health is a key issue for young people. Half of all mental health conditions in adulthood begin before the age of 14 (4). If disorders persist through adulthood, they can cause distress and disability, limiting the potential of an individual (5). Negative outcomes associated with mental illness among youth can include school failure, unstable employment and/or poor family and social functioning (5). Research has shown that young people can be hesitant to seek help (6), and it is therefore vital that proactive approaches to promoting mental health, building resilience and reducing the incidence of mental illness in young people are developed.

Encouraging positive coping, mental health and resilience may help prevent the onset of mental health problems and potentially lessen the severity of existing mental health symptoms. Positive coping and resilience support the development of problem solving skills and build and maintain interpersonal relationships, all of which enhance an individual's ability to perform and contribute meaningfully in daily life. Focusing on wellbeing and building resiliency establishes a holistic approach to health, addressing both physical and psychological needs. The World Health Organization defines wellbeing as “*the state in which an individual realizes his or her own abilities, can cope with normal stresses of life, can work productively, and is able to make a contribution to his or her own community.”* Resilience is directly connected to wellbeing. It is about developing the ability to cope with and adapt to new and/or challenging situations. A sense of resilience and positive wellbeing enables a person to approach others and situations with confidence and optimism. Developing strategies that encourage positive wellbeing and resilience with young people is helpful during a period when there are many changes occurring in the transition between adolescence to adulthood.

### Wellbeing and Resilience Theory

The theory of wellbeing, which incorporates the measure of positive emotions, and is defined as an individual's cognitive and affective evaluation of his or her life as a whole (7). This concept is rooted in each person's experiences, their evaluation of these experiences, and includes positive measures, not merely the absence of negative aspects (8). Wellbeing is comprised of three separate but linked constructs: life satisfaction, positive affect and negative affect (8). Life satisfaction includes both global (e.g., school) and specific (e.g., family) cognitive appraisals of an individual's own happiness, and affect describes emotional and mood states that are reasonably stable (9). Wellbeing broadly includes the experience of high levels of positive emotions and moods (e.g., joy, delight), low levels of negative affect (e.g., sadness, anger) and high life satisfaction (7).

Positive emotional functioning or affectivity is associated with better physical health and coping strategies, and this can be improved by environmental influences (10). Therefore, when focusing on enhancing the wellbeing of children, the environment a child is exposed to such as their home and school environments can have a profound impact on their overall emotional functioning and wellbeing. The study of SWB is relevant to the realm of positive education, which reinforces that the skills and states of mind that promote positive emotions and character strengths can also promote learning and academic success (11). Positive education recognizes that the wellbeing of students is of equal priority to academic learning when developing the whole student, and emphasizes the need for school systems to adopt a framework that creates a positive and productive environment for its students (12). The frequent experience of positive emotions has been shown to predict favorable outcomes for individuals across the lifespan. Barbara Fredrickson's Broaden-and-Build theory of positive emotions suggests the experience of positive affect signals wellbeing, and contributes to personal growth and development (13). Fredrickson (14) suggests that discrete positive emotions, for example joy, contentment and love, all share the ability to *broaden* a person's thought-action repertoire, which gives one the opportunity to *build* their lifelong physical, intellectual, psychological and social resources. It is suggested that positive emotions improve psychological resilience (13), and that they lead to better adjustment in the areas of work, relationships, sociability, likability, cooperation, creativity and problem solving (15). High levels of wellbeing have been demonstrated to promote and maintain mental health, can act as a buffer against negative outcomes such as psychological disorders, and may be a protective factor against depression and suicide (16).

Besides the negative impact of poor wellbeing, there is compelling evidence that positive wellbeing is associated with numerous desirable outcomes. Research indicates that children with higher levels of wellbeing are more likely to learn more effectively, have lower levels of absenteeism at school, have more satisfying and successful relationships with others, and better academic engagement (17, 18). The impact of positive wellbeing at childhood can have long term effects as well. Positive wellbeing in childhood are associated with higher satisfaction with work, a high frequency of contact with friends and family, engagement in social activities, and prosocial behavior (19, 20). In one prospective longitudinal study of a nationally representative British 1946 birth cohort study, participants who were rated by teachers on their positive mood and behavior in their early teens were found to have a 62% reduction in the risk of mental health problem later in life even after controlling for sociodemographic factors, cognition function, and personality (20).

Resilience theorists have provided a theoretical framework to understand how to promote healthy psychological development in individuals that face increased levels of psychosocial adversity (21–25). This theory provides a conceptual roadmap of how researchers and clinicians can work to improve outcomes for young people experiencing mental health problems. It is theorized that youth are not born with innate resilience. Instead, resilience is a developmental process that is facilitated through resiliency promoting activities and events. Masten (22) has named this resiliency as promoting events as “ordinary magic” because they include regular support systems that can be fostered for most developing children through resiliency promoting processes. Resilience promotion can be supported through interventions, such as education and social support, that prevent adversity or reduce its effects and by increasing developmental assets in both an individual and in their ecological systems (22). Completely preventing risk for youth exposed to challenging life events may be a difficult task due to continuance of frequent stressful events. Instead, resilience promotion can be driven by preparing the young person to navigate adversity more successfully.

Resilience occurs when youth engage in positive adjustments to a situation that may include a challenge, risk, and/or adversity (26, 27). In general, the more that a young person are seen as resilient, the better their mental health and wellbeing (28). Youth can experience being resilient when there is a balance between adverse events and protective factors. The presence of protective factors can safeguard young people from the impacts of mental health by potentially “buffering” the impact of the risk factors and reduce the vulnerability of a young person acquiring a mental illness. Protective factors include the young person's own problem-solving skills in response to stressors, psycho-education, and accessible social support from peers and other adults as well as nurture from a primary caregiver (28, 29).

Given the significant role of wellbeing and resilience on various desirable life outcomes, there has been much research investigating ways to promote the wellbeing of youth. There is growing evidence that wellbeing is malleable to change and therefore can be improved (30). Mental health literacy, the knowledge of mental health concepts, adaptive coping skill development, and help-seeking may promote resilience and positive wellbeing in youth (31–33). Furthermore, the educational context such as the school environment and activities offered, social relationships, and means for self-fulfillment has a large influence on youth's overall wellbeing (34).

### Mental Health Literacy and Wellbeing Psycho-Education

Resilience may also be promoted by increasing connections within the adaptive systems in a young person's life (22). One example of this would include participation in groups or classes facilitated by supportive adults, such as a school social worker or school psychologist. Interventions that promote stronger ecological systems allow a developing person to benefit from the adaptive resources of those that surround them. For example, a child may be too overwhelmed to properly follow a family safety plan however, a health care professional can step in and model and implement appropriate crisis intervention strategies. Risk and resilience frameworks should use a systems lens to fully capture the extended network of resilience promoting resources in a developing person's life.

Young people do not have strong mental health literacy and struggle to identify or recognize mental illness disorders or different types of psychological distress (35). In general, young people are poorly informed about mental health (36). The lack of mental health literacy provided to young people is especially salient, as it is during adolescence and early adulthood that health-related behaviors are formed and young people begin to assume responsibility for their health actions (6). Young people also differ in their beliefs and understanding about the nature of mental illness, its causes and the ways to treat mental health difficulties (35).

Some youth may find medically orientated language unhelpful as while it may be knowledge building for some, it can alienate others (37). Gladstone et al. (37) argue that “all knowledge is [not necessarily] power” (p. 1179) and instead suggest that young people require access to different types of knowledge. Notwithstanding the needs of youth, research to date shows that young people usually do not receive developmentally appropriate information (38). Taking into account the findings of research into the effects of wellbeing and resilience, there appears to be a necessity for society to focus not only on recovery and reducing the symptoms of mental illness and distress, but to place equal emphasis on the promotion of positive aspects of mental health and wellbeing of youth. Youth are not always able to access mental health information as they need, at school, home or online. Given that youth spend a substantial amount of time in educational settings, schools have been identified as critically important sites for promoting their wellbeing (39, 40). It is part of the responsibility of schools to promote the health and wellbeing of their students, by enhancing the development of school environments that allow students to feel a sense of safety and belonging, and encourage the development of life-skills and resilience (41).

Within the last decade, digital technologies have been included in the personal, social and occupational routines of young people. More recently, there has been a demand for technology that supports and focuses on youth mental health and wellbeing, evidenced by a recent survey that found around 76% of 525 respondents would use their phone for self-management and self-monitoring of mental health (42). Mental health technology-based solutions co-developed with youth have the potential to play an important role in the future of wellbeing care (43) making support more accessible for youth and potentially reducing barriers to help seeking (44). A review of currently available mental health care technology recommended that mental health technologies are needed to help prevent mental health issues and improve the wellbeing of individuals, and to ease the burden on health, community and school systems (45). There are calls for further understanding of technology and the ways it can support youth mental health (46, 47). Yet, there is a lack of mental health technologies that are specifically developed for and with young people, and little research into the efficacy of this type of technology.

Over half of the population in Australia owns a Smartphone or digital device. Some users check their smartphone, on average 150 times per day, which reflects how applications or platforms can generate and maintain strong habits involving their use (48, 49). While there are interventions available to support vulnerable young people, there is a need for ongoing online support that is interactive, responsive and tailored to the specific needs of youth (1) There are limited effective technologies for young people to use to manage their own wellbeing, for example, using mobile applications or chatbots, remote monitoring or ongoing online access to professional advice (50). The need for better models of online services that improve outcomes and reduce disparities for youth is warranted. Much of the previous work in this area, including development and evaluation of support, has rarely includes the young person's point of view about what they want and need in terms of digital support, such as a chatbot. A chatbot is a digital technology application powered by artificial intelligence that simulates the conversation of another person. A recent scoping review identified 41 chatbots that can be used for mental health, with most implemented in the United States. They are mainly used for therapy, training, and screening, mostly focusing on autism or depression (51). While there are a number of chatbots available none were identified for the youth population and with a focus on mental health literacy and wellbeing. The review suggests chatbots may be useful tools for people with mental health challenges, especially those who are reluctant to seek mental health support due to stigma – an area of particular challenge for young people (51).

The objective of this research seeks to address the accessibility and effectiveness of mental health support experienced by young people by co-developing an online tool that aims to speak directly to youth needs. The overall purpose of this study was to co-develop a chatbot with youth consultation to respond to their wellbeing and mental health needs.

## Methods and Materials

This project is being undertaken across three phases, the paper presenting the preliminary findings from phase one and two:

- **Phase One: Youth and Expert Reference group**: The purpose of the youth and expert reference groups was to meet with young people and experts to inform the personality and development of the mental health chatbot and attain feedback on its improvements throughout its creation. They also provided feedback on the research methods (i.e. questionnaires and interview questions).
- **Phase Two: Chatbot development**: Phase two included a mixed method research design involving a self-constructed questionnaire based on the literature followed by individual semi-structured interviews. Phase two supported the conceptual build of the chatbot with the youth and expert reference group.
- **Phase Three: Gather pilot data**: During phase 3 the chatbot will be tested and used with a wider group of young people to gather feedback of its user ability and applicability. Online surveys will seek to examine youths wellbeing and mental health pre and post chatbot use and interviews to provide feedback about youth experiences of the application to inform future updates, the chatbots personality and to help make the artificial intelligence smarter. Initially, in the pilot phase the chatbot will be implemented in two secondary schools to examine its user ability and also its efficacy and effectiveness in supporting youth with stress and coping. The long-term aim of this project is to provide the chatbot across Australia and to potentially scale it up internationally.

Ethical approval for this study was granted by the relevant University committee.

### Participants

A total of 40 participants accessed and completed the questionnaire through the online survey platform Qualtrics. Eligibility criteria to take part in the study included being a young person aged 15–17 years of age and living in Australia. All participants who completed the questionnaire were invited to individual telephone interviews. Fifteen participants consented to the interviews. Table 1 provides the participant demographics.

**Table 1:** Participant demographics.

**Demographic characteristics**	**Questionnaire participants (*n* = 40)**	**Interview participants (*n* = 15)**
**Age (years)**		
Mean	16.8	16.2
Range		
**Gender**		
Female	30	10
Male	10	5

*All participants were currently enrolled at school*.

### Quantitative Questionnaire Phase

A twenty-item questionnaire was developed, based on current literature and the study objectives. The questionnaire used developmentally and age appropriate language for young people by piloting the questionnaire with the projects youth reference group. After consent was obtained the questionnaire began by asking participants for their demographic information such as age, gender, and if the participant was enrolled in school. The questionnaire asked participants about their interests and the types of technology they use. Then their views of different kinds of online specific supports and/or interventions (i.e., online and digital support such as websites, applications or blogs) was sought regarding support for mental health and wellbeing. Participants were also asked about where they are likely to seek support and if so how or from whom. After each question participants were given an option to mark “other” where they were provided with space to generate their own preferences, ideas and/or suggestions. Each question also provided participants with an open-ended response option to provide additional comments. If participants chose they could receive a $10 gift voucher after completing the questionnaire. Any contact details obtained were stored separately to the questionnaire data and in accordance with University privacy policies.

#### Frequency Statistics

Frequency statistics is the number of times a specific event occurs in a study. In the study, frequency statistics were used in a descriptive manner to identify the amount of responses given by young people on the post intervention satisfaction questionnaire. These responses were summed and tallied to provide the descriptive statistics presented in the findings section.

### Qualitative Interview Phase

At the end of the questionnaire participants were asked if they would like to take part in an individual interview either face to face at the University, via telephone or Skype. The interviews ranged in time between 40 and 60 min. Participants were informed that they could complete the questionnaire without doing the interview, or they could complete both if they chose. All participants needed to obtain parental or caregiver consent before taking part in the interview phase of the project. Similar to the questionnaire there was an option to receive a $10 gift voucher for taking part in an interview.

The semi-structured interview schedule was developed from the literature review and based on the research questions. The purpose of the interviews was to further explore participants' views of using technology for wellbeing support, for example: “What do you think that young people need in terms of online wellbeing support?” Participants were also asked specific questions about what they thought were helpful elements or function of technology applications they found useful. They were given the opportunity to provide input and feedback about what kind of chatbot would be helpful for youths' wellbeing in schools. Interview questions were designed to provide participants with the opportunity to describe their perceptions in a specific area but also provide the opportunity to express their individual views. All interview questions were reviewed by the youth reference group for clarity and developmentally appropriate language. As a participatory qualitative project, the aim of data collection and analysis is not to achieve a statistically representative sample. Instead it is to attain what Glaser and Strauss call ‘saturation of themes’ of data collection until no new themes are generated. Samples sizes have been chosen based on my extensive experience with qualitative projects of this kind.

#### The Interview Process

During the interviews across the various studies, the interviewer first attempted to build rapport with the participants, a stance maintained throughout the interview (52). The interview questions were asked in a general conversational tone to induce a relaxed rather than formal atmosphere (53). At times, the interviewer used probes or prompts after a question to encourage the young person to elaborate (54, 55). Probes or prompts can assist in gaining additional information (54). Please see Table 2 e.g., of the prompts used in the interviews.

**Table 2:** Examples of Probing Techniques Used in the Interviews (54).

**Type of probe or prompt technique**	**Description of probe or prompt**
Silent	The interviewer remains silent and allows the participant to think aloud.
Echo	The interviewer repeats the participant's point, encouraging him or her to develop it further.
Verbal agreement	The interviewer expresses interest in the participant's views with the use of phrases such as “uh-huh” or “yes, okay.”
“Tell me more”	The interviewer clearly asks the participant to expand on a particular point or issue—without the use of echoing.
Long question	The interviewer asks a lengthier question that also suggests that a detailed response is sought.
Leading	The interviewer asks a question that encourages the participant to explain his or her reasoning.

Once both written consent from the young person and their parent or primary care giver was received, all 15 interviews were conducted over the telephone and audio-recorded for later transcription. Interview transcriptions were analyzed using thematic content analysis (56). Thematic analysis is a systematic means of describing and organizing phenomena—a useful process for the exploratory phase of broad research tasks (56, 57). Through this process, common threads of rich and in-depth data were searched for across the interview transcripts. The common threads of data were analyzed by identifying, exploring and reporting patterns (themes) within the data (58). Through the thematic analysis process, data from each study were analyzed using a coding system whereby labels were attached to lines or paragraphs of data to describe the data at concrete and more conceptual levels (59).

Given that the analysis was directed by specific constructs around young people's experiences and views, the analysis procedure used in this thesis can be defined as a “bottom-up” approach (60). A bottom-up approach was more beneficial in extrapolating young people's perspectives than a top-down approach driven by theory analysis. A bottom-up approach is tied closely with thematic analysis in that it is a systematic means of describing and organizing information, whereby data are systematically labeled and classified (56). More specifically, a six-phase process developed by Braun and Clarke (56) was conducted to analyse the data in these studies thematically as identified in Table 3 below. All open-ended questions in the questionnaire were analyzed using thematic analysis.

**Table 3:** Description of Qualitative Analysis Phases [adapted from Braun and Clarke (56)].

**Thematic analysis phase**	**Description of phase**
1. Familiarization with the data	The researcher engaged with and became familiar with the data, including reading and re-reading, taking note of any observations.
2. Coding	Labels were created for significant features of the data that were relevant to the overarching research question directing the analysis, thus aimed to encapsulate a semantic and conceptual reading of the data.
3. Searching for themes	A search for meaningful patterns within the data was conducted whereby themes were interpreted and constructed to be collated into coded data.
4. Reviewing themes	Themes were verified to fit in relation to the coded extracts and the full dataset. Themes were placed together, split into multiple other themes, or abandoned all together.
5. Defining and naming themes	A detailed analysis of each theme was completed that involved pinpointing the core meaning of each theme and then providing an explanatory name for each theme.
6. Writing up a report	This consisted of bringing together the analytic narrative and data extracts that informed the researcher of a clear story about the data, and related back to the existing literature.

#### Trustworthiness

A central issue with qualitative research is the trustworthiness of its processes (also known as credibility or dependability) (58). The approaches used in the studies in the thesis to ensure data trustworthiness included (a) member checks (61), (b) inter-rater reliability (62), and (c) peer debriefing (57).

#### Member Checks

Member checks (also known as respondent validation) were employed whereby interview participants were given the opportunity to view their individual transcripts and change anything they thought was incorrect or potentially identifiable, or to add anything they considered important (61). Participants were asked at the end of the telephone interview if they would like a copy of the transcript. Two of the 15 participants asked to receive a copy of their interview transcript. The researcher did not contact participants who chose not to receive a copy of their transcript. However, they were provided with the researcher's contact details and were able to contact the researcher should they change their mind. Participants were given a month to make any changes to their transcripts before the researcher started to analyse the interview data qualitatively. Two weeks before the month time frame ended, the researcher contacted the two participants via email to check whether there were any changes and to remind participants that there were 2 weeks remaining to send through any amendments to the transcript. None of the two participants sent through any changes to their transcripts.

#### Inter-rater Reliability

A process of inter-rater reliability was used. In this thesis, another researcher re-coded ~30% of the interview transcripts in each study to ensure consistency and reliability of the extrapolated themes (62). The following process was undertaken: (a) each researcher independently coded a transcript into main themes and subsequent subthemes, (b) the two researchers compared their findings through discussion, and finally (c) they discussed any disagreements in the themes to reach a final version (63). There were some disagreements between the researchers regarding one main theme and two subthemes. The researchers managed these disagreements through the process of “negotiated agreement” (64) in a process of discussion and revision of the extrapolated themes by re-reading and reviewing the interview transcripts together. None of the themes generated were irreconcilable.

#### Peer Debriefing

Peer debriefing is a process that can maintain the credibility and validity of research (57, 65). Peer debriefing was implemented using a personal research diary of the researcher's reflections and field notes. A peer, who was another researcher, was invited to read the research diary and provide commentary and critical questions about the decisions and processes implemented by the lead researcher. The goal of the peer debriefing was to provide an analytical probe to explore any biases, viewpoints or assumptions held by the lead researcher (66). For example, the peer asked a question relating to the researcher's use of certain probes during the interviews and why the researcher used certain questions in response to the participant's answers. The researcher then discussed any underlying thoughts and reasoning as to the directions taken during data collection. This process of questioning and discussing the research method occurred throughout each study. The process encouraged the researcher to become more aware of potential biases held toward the data and in the analysis.

### Youth and Expert Reference Group

During the first phase a youth and expert reference group was established early in the research project to inform the development and ongoing improvements needed of the chatbot. The data gathered through the mixed methods research design and the reference groups informed the build, its content, personality and name by piloting the technology application. Specifically, the youth reference group was established to provide feedback on chatbot to ensure it was engaging for this population. Joint consultation between the key stakeholders groups and with the researchers informed the personality and development of the chatbot. The co-design process included the youth reference group sharing feedback on the chatbots creation and any improvements that could be made of the chatbot. During the collaboration phase, key steps from “The Youth Booth” pilot project were used, such as building rapport and a culture of equal sharing (67). In order for youth to express their experiences, “authentic collaborative partnerships” [(68), p. 215] was critical in building rapport and trust between adults and youth. During phase one The Youth Affairs Council Victoria's Yerp Toolkit (69) established guidelines for engaging and building relationships with young people were used. The Yerp suggests non-jargon language and humor to build trust and rapport with young people. Also using open body language, recommending that being the same height as participants such as crouching down or sitting and smiling is more welcoming, as well as demonstrating active listening through nodding, summarizing key points, and asking questions, to show interest in youths suggestion (69). For example, when youth provided feedback that iniatially interactions with the chatbot was boring, and that it should be non-gendered, the chatbot was updated to include GIFS, and was changed to not be a male or female chatbot. After rapport was established using the Yerp toolkit interviewing techniques to check shared understandings, to provide opportunities to clarify any misunderstandings, and/or to expand upon points youth would like to further share.

The expert reference group consisted of three Educational and Developmental Psychologists, one Forensic Psychologist and one Clinical Psychologist. The experts work in educational settings, hospitals and private practice specializing in supporting the wellbeing and mental health of young people. The experts provided psycho-information regarding the content development such as mental health and educational information, and evidence-based strategies. For example, establishing an alert function to notify a trusting adult if a youth was identified as at risk to themselves or others, the writing of confidentiality limits and consent to engage with the chatbot, and the language to discuss youth concerns.

During all meetings with the reference groups the researchers took notes and used the probing techniques identified in Table 2.

## Results

### Questionnaire Findings

Participants predominantly use Facebook, Reddit, Instagram, Snapchat applications and YouTube, Netflix, news websites. Participants used the internet mainly for social reading and connection. Most participants (*n* = 24) know what a chatbot is and had used one previously. When asked of their thoughts of chatbots participants found them to be interesting and exciting to engage with initially however over time they can become “boring and repetitive”:

“It is fun at first, but it grows boring as it becomes what it is - very robotic. Repetitive, not many good responses, too unreal.” (15-year-old Male)

“That they where undeveloped because it felt like they took random fragments from conversations with other visters and most of the time the bot would say things that did not make any sense” (16-year-old Female).

The most important people in the participants life was their mother (*n* = 13) for some it is both parents (*n* = 4), their friends (*n* = 4), a counselor (*n* = 3), a teacher (*n* = 3), their pet (*n* = 3), a partner (*n* = 3), or an extended family member such as an Aunt (*n* = 2). Eighteen youth engaged in support before with a school psychologist (*n* = 5), a psychiatrist (*n* = 2), counseling (*n* = 4), online emotional support websites such as moodgym, kidshelpline, headspace, smilingmind (*n* = 2), and/or emotional support telephone counseling lines (*n* = 3).

All participants indicated that they get stressed regularly mainly from school (*n* = 10). Specific to school events such as exams, assignments, homework, friendships and relationships were the most stressful. Some youth felt the pressure to do well, thinking of the future, being overly busy or falling behind, and expectations of self and from others as a stress trigger. In response to these stressors youth indicated they would try ignore their stress, keep it a secret, try “deal with on my own,” get annoyed or cry. Two youth found the online emotional support websites helpful to cope with the stress.

Thirty participants indicated that their school talked about mental health and wellbeing through a school education program, from their teacher or in class. Some youth found it “not helpful,” “hard stressful triggering,” and “weird, not everyone understands it in practice so hearing about the theory from an oblivious person is weird having had first hand practical experiences.” While for some youth they found the experience to be informative and eye-opening:

“Informative, covered the basics and what was available however services at the school itself were not mentioned” (15 year old Male)

“It opened my eyes to how prevalent it is; how it could become an issue to anyone. You don't have to have a very severe case in order to have mental health issues - in some regard, we all have mental health issues, permanent or temporary.” (17 year old Female)

“It is a comfortable environment, and teachers will give us some advice on how to deal with stress, etc. Walking out of the talk i felt learnt something.” (16 year old Female).

However, youth suggest that talking about mental health with friends and/or family was most helpful (*n* = 34) because:

“Friends would tell me what they were going through e.g., depression, stress, emotional issues. My mum would talk to me about what depression looks like and asks me how my mental health is.” (16 year old Female)

“A few friends and family of mine have mental health problems so it is easier to talk to them about things because i can sympathize with them” (15 year old Male) “My family was really supportive! At first it was hard for mum and dad to learn how to deal with my anxiety in the right ways, but working with a psychologist and calmly explaining in times really helped. It's all about being open and understanding.” (15 year old Female).

For some though talking with friends or family was “confronting' and that friends or family may “seem really judgmental and see it as a weakness in personality rather than an actual illness and they think people can just get over it, also they don't believe in antidepressants.”

### Interview Findings

While the questionnaire data identified what types of support is accessed and youth preferences the interviews contributed to specifying the chatbot's purpose, goals, context, and its features. Based on the interviews the researchers determined the following elements of the chatbot:

*Purpose:* The Chatbot provides students with psycho-educational and wellbeing support to encourage positive mental health.

*Target Audience*: secondary high school students aged between 15–18 years old who are potentially experiencing issues.

*Context of chatbot use:* The chatbot should be used alongside a wellbeing coordinator, school psychologist, or student services team responsible for student behavior and wellbeing.

*Goal of chatbot:* The chatbot will respond to student issues, a school's virtual wellbeing person. Information provided to students about the chatbot and what it is: “Ash is an online robot who can make jokes, chat about your day or share suggestions whenever you need support. If you are having a bad day at school, unsure of how to cope with learning or with friends then it will give suggestions or strategies that may help. It can also connect you with a supportive teacher or professional if you need an extra hand.”

**Figure 1:**
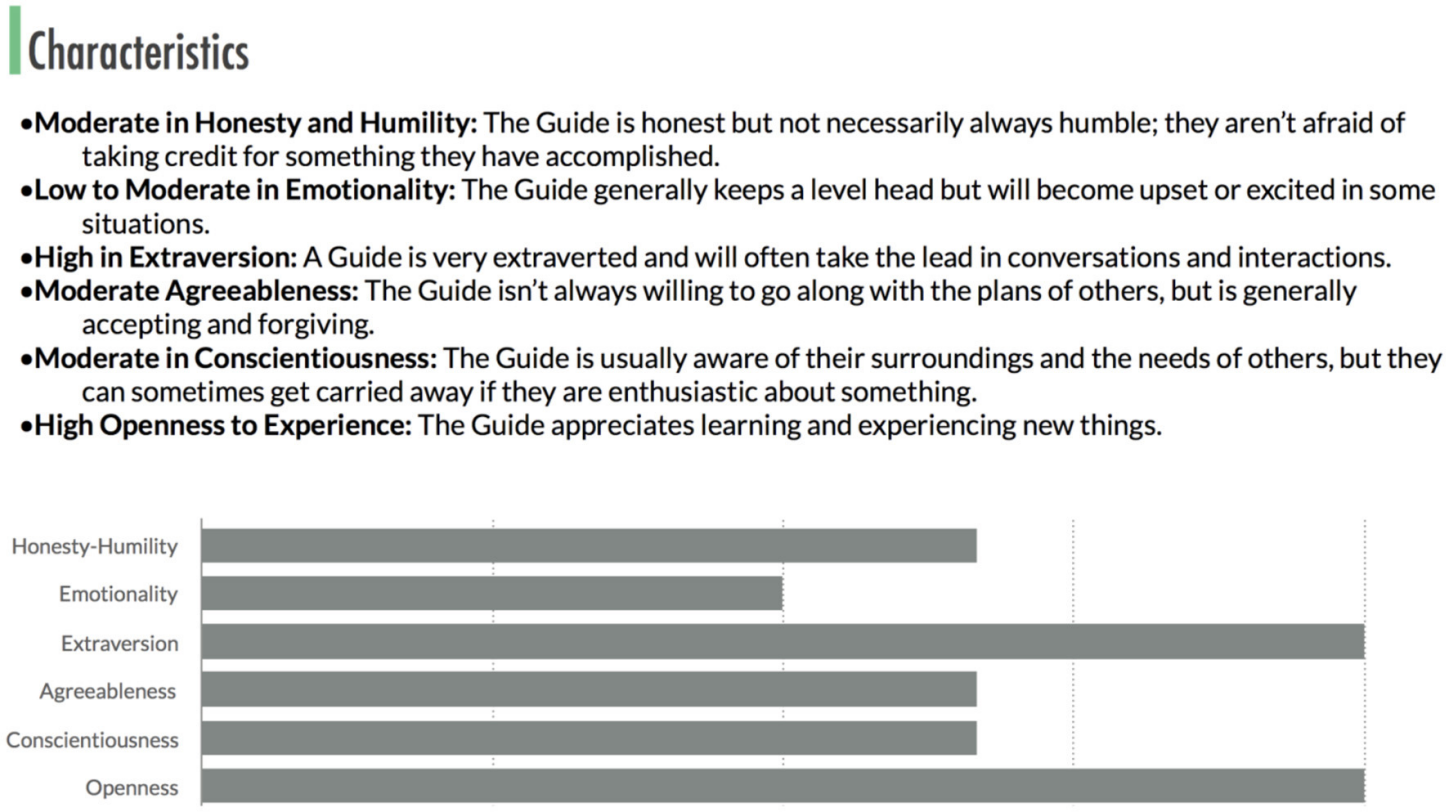
Ash's core personality characteristics.

The interviews also informed Ash's name, gender, feel, personality and the type of “slang” Ash should use. In the interviews participants indicated the following:

*Feel and personality:* Ash is the guide, it is inspiring and charismatic, fun, friendly, empathic, humorous (See Figure 1). It uses emojis, jokes and gifs, a more conversational style of asking questions, storytelling to create a bot persona, when a person is curious it respond one way, when a person is angry it responds another way. When a person is sad, stressed anxious, lonely responds in another way.

Youth also provided a range of specific “slang” words that the chatbot should learn specific to general and Australian teen culture for example footy for football or brekky for breakfast. Several youth suggested a gender neutral chatbot as well as a gender neutral name, several voting for the chatbot to be called “Ash” instead of names such as Bella, Bell, Tim, Xavier etc. Given this feedback the researchers agreed with the name choice of Ash and that Ash would not identify as a specified person rather as a robot. In the initial developments of Ash it was considered to be too feminine according to youths feedback and stereo-typical in that it was a female who was caring and sensitive. Based on this feedback in the interviews Ash does not talk in a gendered way about itself, as youth and expert stakeholders have reviewed and examined the scripts content.

**Figure 2:**
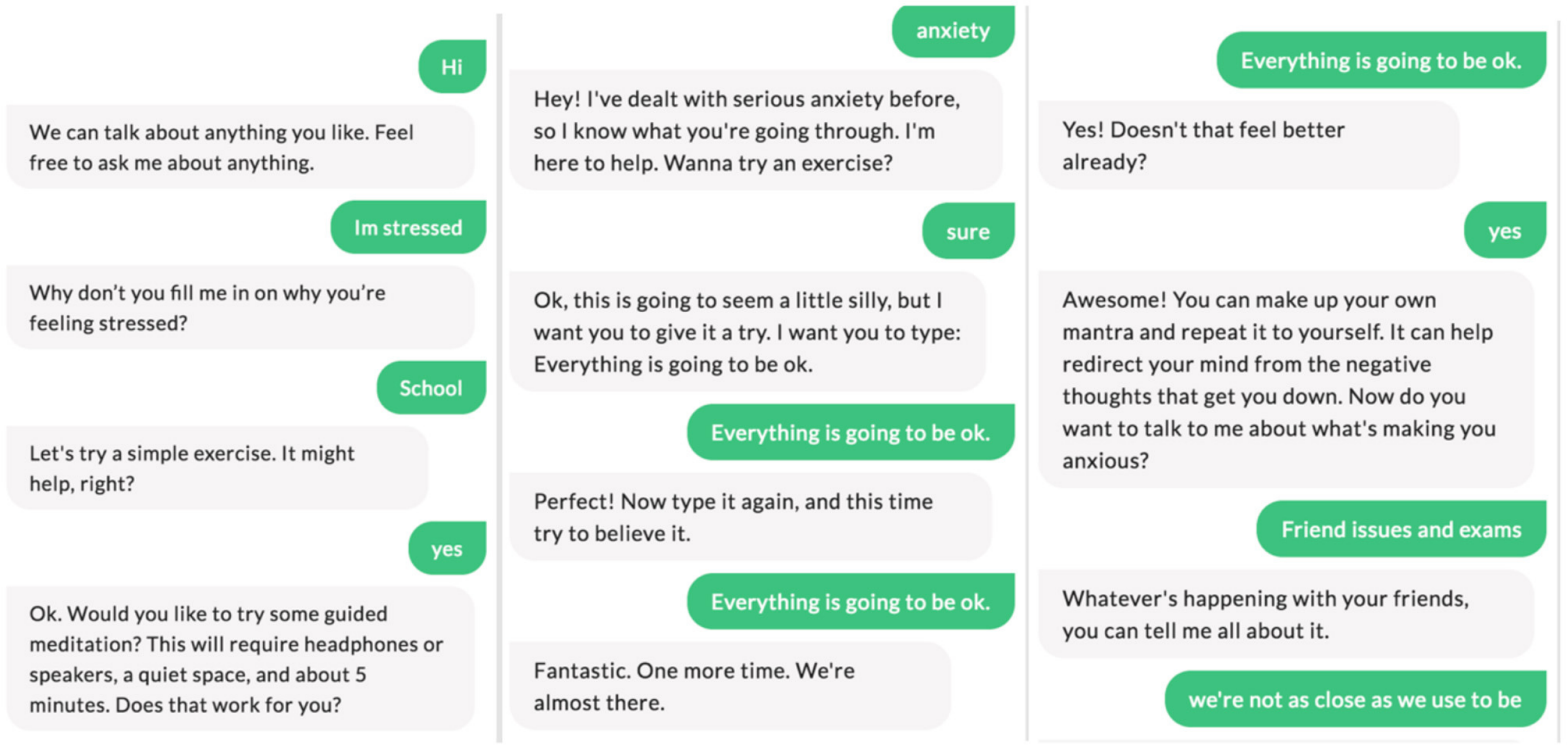
Three images that illustrate the conversation design, flow and content of Ash, and the Chatbot.

*Functions and features*: The chatbot is powered by artificial intelligence - specifically natural language processing. The system is using both pre-scripted and some machine learning that allows it to interpret participant intent and then respond with an answer that is both automatically assembled and hand-written by the researchers, youths and the expert reference group. The chatbot provides all participants with a clear value proposition statement - what can the chatbot do and about confidentiality of information shared. The chatbot is able to handle common phrases/utterances by young people. The implementation for this was be as simple as keyword detection (e.g., hello, thanks, nice, great, swear words), and train a model to detect the longer phrases like how are you, who made you). Whether the chatbot can answer to a simple “thanks” and respond to questions made a difference to the experience and engagement. If a participant wrote “Stop,” “Stop messaging me,” or something similar, the chatbot acknowledged the request and, until otherwise instructed, would stop sending messages. If the chatbot did not understand the intent of the message then it would ask a question or let the participant know they did not understand. A “menu” also was provided to bring up a list of ways the participant can interact with the chatbot, which the participant can access at all times, they can also ask for specific concerns or topics to discuss in the menu bar. The chatbot provided the ability to talk to a person (their support person or a professional) if they have questions or need help. Potentially can be triggered by saying “Contact” and related phrases (if at risk). An example of the conversation design and flow of three different interactions is provided in Figure 2. Edits must be made directly to Ash's script in order to change the manner by which the program operates.

For all participants in the pilot engagement study of Ash a signed consent form was required before interactions could occur. Ash provided the following consent information to be as transparent as possible of the strengths and limits of Ash's ability:

*Consent to engage with Ash the Chatbot:* We are excited for you to use Ash the Monash Chatbot! Welcome! Ash is a robot whose personality is friendly, funny and understanding. Ash is also a good listener and tends to seek out the best in those around it and is a willing communicator who wants to help everyone succeed.

Talking with Ash can help you understand more about yourself. You will have the opportunity to explore how you feel or think about things. Objectives of engaging with Ash can include:

- Feel a sense of support
- Understand more about yourself
- Identify and explore thoughts, feelings and behaviors with strategies that may help

You are welcome to share as much or as little about yourself and ask questions at any time.

Before we begin we need to let you know a few things:

- Ash is not a professional or therapy service
- There are some instances where Ash may have to break confidentiality to keep you safe by making contact with your primary support person.
- Using Ash is voluntary and I can choose to not engage with Ash at any time
- I may choose to nominate and involve a primary family/careers/teacher/other to be my primary contact person if needed. The people I nominate Ash to share information if I'm in danger or may hurt myself or someone else:
  Name:
  Relationship to You:
  Contact Details:
  Signature:
  Date:

The topics covered by the chatbot include both positive and negative: school, family, friends or relationships, the future, religion, books, games, art, sports, exercise and music. In the interviews youth suggested additional topics to be covered by the chatbot, such as information about drugs/alcohol, sexuality, and identity. Suggested strategies for youth include coping strategies, mindfulness and distractions. Also, educational information about how to “shake a bad book” or support for bullying or conflict.

The chatbot also provided links to online recommended resources, such as Reachout a website with supportive strategies to cope and Smiling Mind, an application that provides mindfulness and relaxation strategies. The chatbot Ash provided youth with two key 24/7 professional support both online and telephone that the chatbot connected them with directly, Kids Helpline and headspace.

*Trigger words and sentences:* A list of trigger words and sentences, broken down into 3 (low, medium, high) tier system to trigger to alert system if youth may be “at risk” of self-harm or suicide. These include:

- Low risk: there is no light, I struggle all the time
- Medium risk: I want to hurt myself,
- High risk: I don't want to be alive anymore, I just wish I was dead, I want to cut myself, I want to kill myself

If trigger words were used such as self-harm, suicide, death, dead, kill, die the referral system would be altered. The alert recipient was labeled as a Primary Support Contact who was identified and confirmed during the initial consent process.

## Discussion

Taking into account the findings of research into the effects of mental health and wellbeing, there is a necessity for society to focus not only on the alleviation of mental illness and distress, but to place equal emphasis on the promotion of positive aspects of mental health and wellbeing of developing children and young people. Positive psychologists have striven for interventions of both positive wellbeing and challenges associated with poor mental health in school settings (70). The absence of clinical level of problems is not sufficient to guarantee positive outcomes central to the development of a healthy child (9). For example, on a large number of educational functioning indicators, students with good mental health were found to be more academically successful than their vulnerable peers, who performed lower on measures of reading achievement and exhibited less motivation to regulate their own behavior in order to learn effectively (9). There is increasing recognition that health and educational outcomes are connected, and the school can act as an ideal setting to strive for both (71). As such, the promotion of mental health and wellbeing among children has become a key priority in Australian schools. In October of 2018, the Minister for Education Hon. Dan Tehan, MP, launched the Australian Student Wellbeing Framework. This document is based on evidence that recognizes the strong linkages between student wellbeing and learning outcomes, and includes a set of guiding principles to support schools to build positive learning environments, with advice on developing and implementing policies to support students (The Australian Government: Department of Education and Training, 6). In addition, the Alice Springs Declaration on Educational Goals for Young Australians recognizes the importance of nourishing young people's emotional, mental, spiritual and physical wellbeing, with the development of personal and social capability identified as a key goal in the school Curriculum. In line with this goal, school-wide wellbeing frameworks are developed, with the idea that it is a core responsibility of schools to promote the health and wellbeing of their students, by enhancing the development of school environments that allow students to feel a sense of safety and belonging, and encourage the development of important life-skills and resilience (41). A long-term hope of this project is to potentially use the chatbot in schools or in clinical practice worldwide partnering with international collaborators.

Developing and implementing such programs are based on the notion that schools can provide an environment in which the wellbeing of youth can be improved, to encourage their development into mentally healthy adults. Considering the strong relationship between social settings and emotional wellbeing, and how much young people spend at school, efforts should be made to nurture the emotional wellbeing of students. This can assist families in helping their children become emotionally and socially secure, productive members of the community (71). A review of meta-analyses suggests that programs which promote social and emotional skills and mental health in schools also boost academic attainment (72). Clearly wellbeing is a factor that has been found to consistently predict positive school outcomes. Evidence from experimental studies suggests that programs which aim to promote students' wellbeing and development also have the potential to improve their learning (72), with a positive mood being linked with broader attention as well as more creative and more holistic thinking (73). Yet some schools are not always equipped to provide mental health support. Youth reported here that they are not always able to access support and information about wellbeing as they need. An implication of this chatbot is the potential of technology-based programs to augment these gaps and to offer quality supports and resources for youth through the types of media that youth engage with regularly. For example, the school intranet offers an important avenue for accessing support as it is used routinely by all students for their schoolwork and can offer a private and personalized means of accessing support, in contrast to traditional avenues may be perceived to be less helpful (such as pamphlets) or may contribute to the exclusion of students through identifying them as “different” (such as openly attending appointments with well-being staff). Therefore, it programs which aim to encourage the experience of positive emotions among children at school that can potentially have a profound impact on a child's healthy development.

This project potentially can make important contribution to the engagement of students who may be struggling with mental health or wellbeing while at school. Given many participants here indicate that school is a trigger of their stress it may be key to provide evidence based strategies that are supportive, preventative and encourage positive coping. The chatbot analysis provides an understanding about where artificial intelligence can be effective in its implementation in schools that otherwise might not make best use of providing mental health support. Over the years some researchers believe that chatbots could positively influence people's lives, particularly those experiencing mental health issues (74, 75). They suggest chatbots can be used to aid doctors or clinical practitioners, or in this studies case, it could aid (note replace) school psychologists or wellbeing coordinator/counselors to support youths who are waiting for support (on the referral list) or if they need to access accurate mental health educational support at school.

### Challenges, Cautions, and Potential Limitations

Words of caution and potential limitations include two functional technical challenges when interacting with Ash. There remain two functional technical challenges with Ash that need to be overcome before whole school-wide use can take place. The first is identification of critical words and understanding of these critical words and the second is the generation of responses appropriate to the critical words or lack of critical words. At time of writing Ash tends to move quickly into problem solving mode once an issue has been shared. Ash needs to maintain instead a sense of listening and communicating about the concern at hand rather than trying to find a solution to address the problem. This approach will reduce the generated response being “incorrect” in the conversation. Future iterations of Ash will attempt to have a balance between problem solving and also emphasing and asking further questions to identify the concerns. An additional challenge may be the sample, of the 15 participant who were interviews, only 5 were male and 10 of the 40 surveyed participants were male. Future studies may want to recruit and target males specifically to understand their mental health service experience as well. Also, currently the field of data science and artificial intelligence may not be developed enough to be able to implement all the features suggested by youth and experts during in the consultation phase of the chatbot. For example, although Ash is capable of engaging in conversation but cannot engage with a true understanding. Being upfront in the disclosure of the its capabilities and that, as in the case with Ash, its purpose is not to replace a person to person contact but rather aid school personnel. There are limits to chatbots and artificial intelligence, some anthropologists suggest computers see a reduction of the human being and any life form. Given youth engage regularly in the online world and the waits experienced in schools for wellbeing support there appears to be an opportunity to potentially provide a bridge in conjunction with school personnel.

## Conclusion

Good mental health and wellbeing is about more than just a lack of illness. Focusing on positive coping and building resiliency is important in establishing a holistic approach to health, addressing both physical and psychological experiences of youth. It is clear that young people being aware of mental health and wellbeing literacy has implications for the promotion of healthy well-being and early prevention of mental health disorders. An implication of this project is the potential of technology-based support to augment health service gaps and to offer quality supports and resources for young people through the types of media that youth engage with regularly.

## Data Availability Statement

The raw data supporting the conclusions of this article will be made available by the authors, without undue reservation.

## Ethics Statement

The studies involving human participants were reviewed and approved by Monash University Human Ethical Approvals. Written informed consent to participate in this study was provided by the participants' legal guardian/next of kin.

## Author Contributions

The author confirms being the sole contributor of this work and has approved it for publication.

## Conflict of Interest

The author declares that the research was conducted in the absence of any commercial or financial relationships that could be construed as a potential conflict of interest.
